# Assessment of Supplementation with Different Biomolecules in the Prevention and Treatment of COVID-19

**DOI:** 10.3390/nu16183070

**Published:** 2024-09-11

**Authors:** Anabel González-Acedo, Francisco Javier Manzano-Moreno, Enrique García-Recio, Concepción Ruiz, Elvira de Luna-Bertos, Víctor Javier Costela-Ruiz

**Affiliations:** 1 Biomedical Group (BIO277), Department of Nursing, Faculty of Health Sciences, University of Granada, Avda. Ilustración 60, 18016 Granada, Spain; anabelglez@ugr.es (A.G.-A.); egr@ugr.es (E.G.-R.); crr@ugr.es (C.R.); vircoss@ugr.es (V.J.C.-R.); 2 Biomedical Group (BIO277), Department of Stomatology, School of Dentistry, University of Granada, 18016 Granada, Spain; fjmanza@ugr.es; 3 Institute of Biosanitary Research, ibs.Granada, Avda. de Madrid, 15 Pabellón de Consultas Externas, 2ª Planta, 18012 Granada, Spain; 4 Institute of Neuroscience, University of Granada, 18016 Granada, Spain

**Keywords:** COVID-19, vitamin D, antioxidant vitamins, melatonin, lactoferrin

## Abstract

Consequences of the disease produced by the severe acute respiratory syndrome coronavirus 2 (SARS-CoV-2) have led to an urgent search for preventive and therapeutic strategies. Besides drug treatments, proposals have been made for supplementation with biomolecules possessing immunomodulatory and antioxidant properties. The objective of this study was to review published evidence on the clinical usefulness of supplementation with vitamin D, antioxidant vitamins (vitamin A, vitamin E, and vitamin C), melatonin, lactoferrin and natural products found in food (curcumin, luteolin, ginger, allicin, magnesium and zinc) as supplements in SARS-CoV-2 infection. In general, supplementation of conventional treatments with these biomolecules has been found to improve the clinical symptoms and severity of the coronavirus disease (COVID-19), with some indications of a preventive effect. In conclusion, these compounds may assist in preventing and/or improving the symptoms of COVID-19. Nevertheless, only limited evidence is available, and findings have been inconsistent. Further investigations are needed to verify the therapeutic potential of these supplements.

## 1. Introduction

SARS-CoV-2 is the causal agent of COVID-19, which was declared a pandemic by the WHO on 11 March 2020. The initial strain of SARS-CoV-2 and its multiple variants have infected millions of individuals over the past few years, causing millions of deaths worldwide [1]. In this sense, the WHO describes more than 700 million infections and around 7 millions of deaths by 2024 [2].

SARS-CoV-2 is a betacoronavirus that enters the host cell through a glycoprotein in the form of a viral spike or peplomer, giving it the appearance of a crown. The spike is formed by two subunits known as S1 and S2. Subunit S1 binds to the receptor of angiotensin-converting enzyme 2 (ACE2) on the host cell surface, allowing its recognition and binding to the specific cell receptor of the host. Subunit S2 permits the fusion between the viral and cell membranes [3,4].

The expression and distribution of ACE2 in the organism indicate the potential routes of SARS-CoV-2 infection because ACE2-expressing cells can act as susceptible viral targets, and the viral replication cycle begins in these cells. Expression of this enzyme is elevated in the human organism, being detected in renal, cardiovascular, gastrointestinal, and lung tissue cells, among others (in oral/nasal mucosa, the nasopharynx, the lung, the stomach, the small intestine, the colon, skin, lymph nodes, the thymus, bone marrow, the spleen, the liver, kidneys, and the brain) [5,6,7]. In this way, the signs and symptoms of COVID-19 are related to cell damage produced by the infection of tissues and the stimulation of the host immune system to activate anti-virus mechanisms [8]. Furthermore, epigallocatechin-3-gallate, a multi-species anti-SARS-CoV-2 therapeutic candidate derived from green tea, is highly effective in suppressing viral replication by interfering with the binding between ACE2 and protein spike. In addition to this example, there are currently more biomolecules that could act as an effective treatment for this disease [9].

The most common symptoms of COVID-19 are fever, coughing, fatigue, and loss of the sense of taste and/or smell, with the possible association of muscle and/or throat pain, headaches, hemoptysis, and/or diarrhea. One of the most worrying signs is dyspnea, which may indicate pulmonary complications such as pneumonia, with the possible development of acute respiratory distress syndrome. Death can result from the severity of these complications and from the presence of cardiac involvement and/or coagulopathy. Around one-fifth of patients develop severe disease, while most patients have only mild or no symptoms [10,11]. However, during the early pandemic and periods of social restrictions, a slight but consistent decline in mental health, especially depression, has been observed in the general population and among individuals with chronic somatic disorders [12].

The risk of severe COVID-19 disease has been associated with the viral load, patient age and sex, genetic and environmental factors, and the history/presence of cardiovascular disease, diabetes mellitus, and chronic obstructive pulmonary disease, among others [10]. When the course of the disease is torpid, it is possible to detect the involvement of numerous systems/organs, including disorders in the lungs, with dyspnea and pneumonia; kidneys, with possible onset of acute renal failure; the heart, with arrythmias and infarction; the liver; digestive systems, with abdominal pain, diarrhea, and nausea; the vascular system, with the emergence of coagulopathies; the neurological system, with migraines, convulsions, and even strokes; and the immune system, with the development of an intense inflammatory response (“cytokine storm”), which can lead to multiple organ dysfunction syndrome and death [11,13,14].

The rapid expansion of COVID-19 disease and its severe consequences have prompted widespread research into its prevention and treatment. Besides pharmacological approaches (e.g., corticosteroid and biological therapies), there has been special interest in biomolecules with immunomodulatory and antioxidant properties, such as vitamin D, antioxidant vitamins, lactoferrin, and melatonin [15,16,17,18,19,20]. The aim of this study was to examine published evidence on the effectiveness of different biomolecules to treat and/or prevent COVID-19.

## 2. Results

The following results provide information on the clinical usefulness of supplementation with vitamin D, antioxidant vitamins (vitamin A, vitamin E, and vitamin C), melatonin, and lactoferrin (Table 1).

### 2.1. Vitamin D

Vitamin D is a fat-soluble steroid hormone [88]. The main source of vitamin D in humans is endogenous, deriving from photochemical synthesis by solar irradiation of 7—dehydroxycholesterol on the skin, producing cholecalciferol or vitamin D3. The diet supplies vitamin D in the form of ergocalciferol or vitamin D2, largely from the consumption of dairy products or fish liver oil [89,90]. However, the vitamin concentration from this exogenous source can often fall below daily replacement requirements. Both cholecalciferol and ergocalciferol require two hydroxylations in the liver and kidney for transformation into calcitriol (1,25 (OH)2D3), their active form [91].

Vitamin D has long been known to play a role in calcium regulation, bone health, and the prevention of rickets [92]. More recent investigations have highlighted its influence on immune system cells and its important anti-inflammatory function, mediated by immunological mechanisms [93,94]. Vitamin D is also an epigenetic regulator of more than 2500 genes [95] related to cancer [96,97], diabetes mellitus, acute respiratory tract infection [98], and autoimmune diseases, such as multiple sclerosis [99,100].

The immunological role of vitamin D in respiratory infections is based on suppression of the adaptive immune response of respiratory epithelial cells [101,102]. In vitro studies and clinical trials of vitamin D supplementation have evidenced the inhibitory action of vitamin D against syncytial respiratory virus, influenza, and SARS [101,103] and against non-respiratory viruses such as HIV-1, hepatitis C virus, and dengue virus [104,105,106]. Viral evasion mechanisms are mediated by a decrease in the proliferation of T cells and consequent transformation of T-helper 1 (Th1) cells into T-helper 2 (Th2) cells [107]. In this manner, a decrease in Th1 cells reduces the production of proinflammatory cytokines with cytotoxic activity. Vitamin D may also affect T cell maturation by diverting the development of T-helper 17 (Th17) cells towards anti-inflammatory regulatory T cells [102,107]. Thus, vitamin D can reduce levels of proinflammatory cytokines (interleukin-1 [IL-1], IL-6, IL-12, tumor necrosis factor-α [TNF-α], and IL-17) and simultaneously increase levels of the anti-inflammatory cytokine IL-10 [101,102]. Downregulation of inflammatory cytokine expression inhibits the differentiation and activation of various types of immune cells and could possibly prevent immunomediated lesions [102]. Hence, vitamin D plays a complex dual role in immunopathology through its actions on cytokine regulation and T cell differentiation [101,102].

Another antiviral mechanism of vitamin D derives from its capacity to increase the secretion of antimicrobial peptides that influence the viral replication cycle [108,109,110]. Schögler et al. observed that in vitro vitamin D supplementation increased levels of antimicrobial peptide LL-37 and reduced the viral load in cystic fibrosis airway epithelial cells infected with rhinovirus 16 [111].

It was recently reported that vitamin D can downregulate ACE2 receptors and may possibly have certain protective effects against viral infections such as COVID-19, whose entry at intracellular level is mediated by this enzyme [112]. 

#### 2.1.1. Vitamin D and Prevention of Infection by SARS-CoV-2

Since the start of the COVID-19 pandemic, authors have proposed a link between vitamin D and the risks of infection or severe disease, based on data from populations characterized by vitamin D deficiency due to their geographic location, vitamin D-poor diet, or lack of vitamin D supplementation [21].

Hypovitaminosis D is widespread, especially in southern European countries, where COVID-19 has had a major impact. This disease can result from inadequate solar exposure without vitamin D-enriched food or supplementation. Low vitamin D levels are also more likely at an advanced age, when there is a higher risk of complications associated with COVID-19, especially in the presence of diabetes mellitus, obesity, or other comorbidities [22]. A meta-analysis published in 2021 suggested an association between hypovitaminosis D and an increased susceptibility to infection by SARS-CoV-2 [23].

Vitamin D deficiency has also been associated with hypocalcemia, which was diagnosed in 80% of patients with symptomatic COVID-19 attended in a hospital emergency department in Italy [24,25]. A study of over-50-year-olds from Asia, Europe, and USA described an association between vitamin D deficiency and a greater risk of symptomatic SARS-CoV-2 infection. It also reported lower vitamin D levels in patients with a positive versus negative SARS-CoV-2. The authors therefore concluded that low vitamin D levels might be linked to a higher risk of SARS-CoV-2 infection, in agreement with the results of various meta-analyses [23,26]. A retrospective one-year study in the USA on the serum vitamin D levels in 190,000 patients observed a higher frequency of SARS-CoV-2 infection in patients with versus without vitamin D deficiency [27]. In the same line, a double-blind randomized clinical trial demonstrated an association between vitamin D and a lower risk of this infection [113]. However, a study of around 41,000 serum calcifediol measurements found no direct relationship between vitamin D levels and the risk of infection by SARS-CoV-2 [28]. Furthermore, a randomized clinical trial in the UK with a sample of 6200 patients found that vitamin D supplementation did not reduce the risk of acute respiratory infection, including COVID-19 [29].

#### 2.1.2. Vitamin D and Complications Associated with SARS-CoV-2 Infection

Various studies have described vitamin D as a biomolecule that can make an important contribution to attenuating the severe complications of SARS-CoV-2. Researchers have reported an inversely proportional relationship between serum vitamin D levels and the severity of COVID-19 disease in terms of a higher mortality risk and the need for intensive care unit (ICU) admission and mechanical ventilation [26,30,31]. Regarding the severity of lung involvement, one retrospective study observed vitamin D deficiency in 55% of patients in radiological stage 1 of lung involvement due to pneumonia and in 74% of those with stage 3 involvement [32].

Another study reported that COVID-19 severity was related to decreased vitamin D levels, which were in turn associated with increased ACE2 and IL-6 levels [33]. A longitudinal study found that optimal vitamin D levels protected against the development of severe disease [34].

A relationship has also been observed between vitamin D deficiency and COVID-19-related mortality [35,36,37]. The prognosis was found to be even less favorable in patients with this deficiency and comorbidities, such as diabetes mellitus and/or obesity [38]. In contrast, a Mendelian randomization study with a sample of 443,734 individuals found no association between vitamin D levels and COVID-19 severity [39]. In the same line, Domazet Bugarin et al. did not observed statistically significant difference on the number of days on respiratory support, even though the trial lacked sufficient power for the main outcome [40]. Another randomized controlled trial observed that vitamin D did not contributed to a shorter recovery time in patients who still tested positive by RT-PCR on the 14th day [41]. Lastly, Dilokpattanamongkol et al. showed that the intervention group evidenced a more substantial reduction in the pneumonia severity index from enrollment to discharge. Moreover, patients with C-reactive protein levels greater than 30 mg/L also experienced a significant decrease [42].

Findings to date generally indicate an inverse relationship between vitamin D levels and risks of infection by SARS-CoV-2 and of severe disease. Populations should be informed about the potential benefits of vitamin D in protecting against infections, including infection by SARS-CoV-2, and should be told about the ways from which it can be obtained.

### 2.2. Antioxidant Vitamins

Vitamins A, E, and C have been proposed as candidates for the treatment of COVID-19 disease due to their antioxidant properties [18]. Their usefulness has been explored in multiple clinical trials.

#### 2.2.1. Vitamin A

Vitamin A is a fat-soluble micronutrient known also as retinol or retinoic acid. It has antioxidant and anti-inflammatory activities, among other properties, and it has demonstrated beneficial effects on immune and respiratory system functioning [114]. Vitamin A is important for the maintenance of epithelial tissues and in the physiological secretion of mucosal epithelia. It also plays a key role in the immune system, improving the number and functional capacity of innate immune cells and promoting immunoglobulin synthesis [115,116,117]. Consequently, vitamin A deficiency is associated with a higher risk of infection [118,119], reducing Th2-mediated antibody responses, hampering the regeneration of infection-damaged mucosae, and impairing the function of neutrophils, macrophages, and natural killer cells [114,118]. In a study of 155 patients diagnosed with COVID-19, 37% were vitamin A-deficient (serum level <0.343 mg/L), and serum levels were 23% lower in patients with severe symptoms than in asymptomatic patients [120]. The authors suggested that vitamin A depletion in serum results from its utilization in the innate immunity system, mediated by neutrophils, dendritic cells, monocytes, and macrophages. Depletion of this vitamin would therefore involve a shift from an innate response to an adaptive response that does not require retinoic acid [121]. These authors also reported significantly lower mean serum vitamin A levels in 27 ICU patients with severe COVID-19 (0.37 mg/L) than in 25 asymptomatic patients (0.52 mg/L), proposing that low levels at the onset of infection might contribute to an increase in inflammation and a severe disease pathogenesis [45].

Various authors have evaluated the effect of vitamin A supplementation on the severity of COVID-19 disease. One clinical trial [43] described significant improvements in fever, fatigue, weakness, and body pain after 10 days of supplementation with 25,000 IU of vitamin A in comparison to controls treated with hydroxychloroquine alone. Among the clinical parameters considered (C-reactive protein [CRP], white blood cells [WBCs], lymphocytes, erythrocyte sedimentation rate [ESR], creatine phosphokinase, creatine test, and liver function tests [alanine aminotransferase and aspartate aminotransferase]), supplementation reduced CRP and WBC counts and increased the lymphocyte count, with no other significant changes [43]. These effects can be attributed to the fact that retinoic acid signaling starts during inflammation development, when the differentiation, maturation, and function of innate and adaptive immune system cells are regulated [119]. In this regard, vitamin A supplementation has been found to reduce complication and mortality rates in patients with measles, Ebola virus, and HIV-1 infection; hence, its deficiency has been associated with a greater susceptibility to infections and with an increase in their severity and duration [122]. In addition, a recent meta-analysis concluded that vitamin A supplementation can improve the effectiveness of conventional therapy in children with pneumonia [123]. In fact, vitamin A deficiency is highly frequent in children with pneumonia, whose incidence in pediatric populations might be related to serum levels of vitamin A [123].

However, a pilot clinical trial found no difference in clinical response, ICU admission rate, or need for mechanical ventilation between hospitalized patients with COVID-19 receiving supplementation with 50,000 IU of vitamin A and those receiving a placebo [44]. 

Studies have generally shown that retinol can have protective and adjuvant effects on the development of COVID-19 and the prognosis of infected patients, but further research is required to confirm and elucidate these observations.

#### 2.2.2. Vitamin E

Vitamin E, or α-tocopherol, is a fat-soluble micronutrient that is essential for organism homeostasis. It possesses antioxidant activity, neutralizing peroxyl radicals and breaking the chain reaction of lipid peroxidation in cell membranes and lipoproteins [124,125]. Vitamin E also modulates immune and inflammatory responses through its participation in the activation, development, and functioning of dendritic cells, macrophages, T cells, B cells, and natural killer cells [126,127]. Although only a few studies have been conducted in humans, some have suggested that vitamin E supplementation can reduce the incidence of respiratory infections in the elderly [127,128].It has also been reported that antioxidant therapy with vitamin C and E supplementation might reduce the risk of cardiac complications in patients with COVID-19 [129]. 

Various studies have described low serum vitamin E levels in patients with COVID-19, except in those receiving supplementation [120,130]. It has therefore been proposed that vitamin E supplementation may be of benefit in the treatment of this disease [120,131].

In a study of 110 adult patients hospitalized in ICU with moderate or severe pneumonia due to COVID-19, 22 patients took a capsule of vitamin E (α-tocopheryl acetate) at a dose of 400 IU (equivalent to 800 mg) plus a 400 mg pill of anti-inflammatory pentoxifylline every 12 h for 5 days [46]. This treatment was found to reduce plasma lipid peroxidation levels, increase total antioxidant capacity, and exert a synergic effect on the inflammatory response, with a reduction in the inflammation markers IL-6, CRP, and procalcitonin (PCT). The authors concluded that the combined treatment of vitamin E and pentoxifylline can serve as coadjuvant treatment in patients with severe COVID-19 [46].

In a randomized trial by Beigmohammadi et al. [47], 30 ICU patients with COVID-19 were administered with the following vitamin supplements for 7 days: 25,000 IU of vitamin A once a day, 300 IU of vitamin E twice a day, 500 mg vitamin C four times a day, a vial of vitamin B complex [thiamine nitrate 3.1 mg, sodium riboflavin phosphate 4.9 mg (corresponding to vitamin B2 3.6 mg), nicotinamide 40 mg, pyridoxine hydrochloride 4.9 mg (corresponding to vitamin B6 4.0 mg), sodium pantothenate 16.5 mg (corresponding to pantothenic acid 15 mg), sodium ascorbate 113 mg (corresponding to vitamin C 100 mg), biotin 60 μg, folic acid 400 μg, and cyanocobalamin 5 μg] once a day, and 600,000 IU of vitamin D once during the study period. This treatment was found to reduce disease severity and serum levels of the inflammatory markers ESR, CRP, IL6, TNF-ɑ, and interferon-γ. Serum levels of all vitamins were significantly increased after 7 days of treatment, and there was a significant reduction in the rate of prolonged hospitalization of more than 7 days; however, there was no improvement in the mortality rate in comparison to controls receiving no vitamin supplementation [47].

Only limited evidence has been published on the usefulness of vitamin E as therapeutic agent against COVID-19, and further research is warranted.

#### 2.2.3. Vitamin C

Vitamin C, or ascorbic acid, is a water-soluble micronutrient known for its anti-inflammatory properties and capacity to eliminate free radicals. It is also involved in catecholamine and steroid synthesis, iron absorption, and collagen synthesis. In relation to the antimicrobial and immunomodulatory properties of vitamin C, it regulates the release of proinflammatory and proapoptotic cytokines (e.g., IL-6 or TNF-α) and inhibits the activation of nuclear factor kappa-light-chain-enhancer of activated B cells (NFκB) [48,132]. One advantage of vitamin C over other antioxidant vitamins is that it is water soluble, and excess amounts are therefore excreted in the urine, avoiding hypervitaminosis [49].

In regard to immunological mechanisms, it has been demonstrated that high doses of vitamin C can regulate the proliferation and function of T lymphocytes, B lymphocytes, and natural killer cells [133]. It may therefore be a useful approach to the cytokine storm responsible for a worsening of patients with COVID-19 [134]. It has been observed that the bioavailability of vitamin C is very low in patients with COVID-19, likely attributable to its antioxidant activity, leading some authors to recommend its administration as a supplement regardless of its therapeutic potential [49].

Inconclusive and contradictory findings have been published by studies on the usefulness of vitamin C supplementation against COVID-19 at different stages and severity levels. Majidi et al. reported that administration of 500 mg vitamin C for 14 days almost doubled the survival of ICU patients with severe COVID-19, although it had no significant effect on blood biochemistry values [50]. In contrast, one study of patients in a similar condition found that high doses of vitamin C had no impact on partial oxygen saturation, length of ICU stay, or mortality rate [51]. Al Sulaimán et al. administered low vitamin C doses to patients with severe COVID-19 in the ICU and found no reduction in the mortality rate, although there was a lower frequency of thrombosis in comparison to controls [52]. In another study of hospitalized patients with moderate or severe COVID-19, treatment with very high doses of vitamin C doses (2 g/6 h) for 5 days improved partial oxygen saturation, respiratory rate, and lung damage detected by computed tomography [53]. Another randomized controlled trial observed that after 28 days of supplementation with l-arginine plus vitamin C there was a significant increase in serum l-arginine concentrations and the l-arginine/ADMA ratio compared to the placebo group [58].

Heterogeneous results have also been obtained in patients with mild or moderate COVID-19. In one trial, application of an oral spray based on artemisinin, curcumin, incense (Boswellia), and vitamin C improved the National Early Warning Score (NEWS2), partial oxygen saturation, need for oxygen therapy, and fever in patients with moderate or mild COVID-19 in comparison to those receiving placebo [135]. However, a study that compared the antioxidant effects of vitamin C, melatonin, and placebo concluded that vitamin C had no significant impact on the symptoms of patients with mild COVID-19 [54]. Inconclusive results have also been obtained in patients with mild COVID-19 treated in an outpatient setting. In one study, the combination of vitamin C and zinc significantly increased the humoral immune response to the virus [56]. However, Thomas et al. [55] observed no significant improvements in the symptoms of patients (n = 214) after 10 days of treatment with zinc gluconate, vitamin C, zinc gluconate plus vitamin C, or the standard approach. 

Finally, it was reported that treatment with vitamin C and L-arginine for 28 days significantly improved gait, muscle strength, endothelial function, and fatigue in patients with persistent COVID-19 [57].

Evidence remains inconclusive on the usefulness of vitamin C in patients with COVID-19, and further in-depth research is needed to verify its potential benefits.

### 2.3. Melatonin

Melatonin, or *N*-acetyl-methoxy-tryptamine, is a primary hormone released by the pineal gland, mainly at night, although it is also synthesized in lymphocytes, bone marrow, eyes, and the gastrointestinal tract [59,136]. *N*-acetyl-methoxy-tryptamine is derived from tryptophan, an amino acid present in numerous foods, including wheat, milk, rice, meat, beer, coffee, wine, and fish [137].

The primary function of melatonin is to act as an endogenous circadian rhythm synchronizer and sleep regulator [138], but it has also been attributed with anti-inflammatory, antioxidant, and immunoregulatory properties [139,140], and it may reduce the risk of thrombosis [141]. Given these positive effects, melatonin has been studied in various randomized controlled clinical trials as a coadjuvant in the treatment and prevention of COVID-19 [54,59,60,61,62,63,64,65]. 

In a randomized controlled clinical trial on its preventive effects, healthcare professionals with high exposure to SARS-CoV-2 received prophylactic treatment with 2 mg/day oral melatonin for 12 weeks; however, there was no significant improvement in the rate of infection by the virus [59]. In contrast, Ameri et al. found melatonin significantly improved clinical status (*p* < 0.05) (mortality rate, number of days to hospital discharge and time to clinical status improvement) with a safe profile in patients with severe COVID-19 pneumonia [66]. There has been more research on the therapeutic potential of this hormone.

Ameri et al. studied the effectiveness and safety of oral melatonin in patients with COVID-19 who had severe pneumonia, obtaining significant reductions in the mortality rate (67% in melatonin-treated group versus 94% in controls), need for mechanical ventilation (51.4% vs. 70.9%), and length of hospital stay (15 vs. 21 days). Farnoosh et al. found that supplementation of patients hospitalized for COVID-19 with low-dose oral melatonin (3 mg 3 times/day) significantly improved their symptoms (coughing, dyspnea, and fatigue) and reduced their CRP levels in comparison to controls receiving the standard treatment alone. Similar results were obtained in a double-blind clinical trial of high-dose melatonin (21 mg/day) as coadjuvant therapy in intubated patients, and the significant decreased CRP levels in melatonin-treated patients was interpreted as a possible a reduction in inflammatory response; however, there were no differences with controls in the mortality rate or duration of mechanical ventilation [62]. Likewise, an observational retrospective study of adults hospitalized for COVID-19 found that the mortality rate was not affected by the receipt of 2.1 mg of melatonin for a mean of 15 days [61]. However, the administration of a higher dose (10 mg/day) of oral melatonin significantly reduced the frequency of thrombosis and sepsis and the mortality rate in hospitalized patients with COVID-19 [65].

A comparative study in patients with moderate COVID-19 found no significant symptom improvement in those treated with oral vitamin C (1000 mg/day) versus placebo but significant improvements in symptoms and quality of life in those treated with oral melatonin (10 mg/day) versus vitamin C or placebo [54]. Another study in patients hospitalized for COVID-19 observed significant improvements in sleep and oxygen saturation in those receiving 3 mg oral melatonin before sleeping [64].

There is some evidence supporting the usefulness of oral melatonin to improve the symptoms of COVID-19. However, further clinical trials are required to test the efficacy of melatonin in the prevention and treatment of this disease.

### 2.4. Lactoferrin

Lactoferrin (Lf) is an iron-binding glycoprotein of 80 kDa and 703 amino acid residues secreted by exocrine glands and neutrophils [142]. Structurally, it is a polypeptide folded into two symmetrical lobules that can transport iron in blood and serum [143]. Lf is found in humans (hLf) and some other mammals, including bovines (bLf). hLF and bLF have similar sequencing and function and are of interest for their role in innate immunity. Both have been attributed with anti-inflammatory, antiviral, and immunomodulatory properties, which are especially relevant for the treatment of infectious processes such as SARS-CoV-2 [144,145,146].

It has been observed that SARS-CoV-2 tends to infect cells expressing the ACE2 gene and gains access to cells through the fusion of plasmatic membranes or endocytosis. Another binding factor is heparan sulfate proteoglycans (HSPG), which facilitates binding of the virus to ACE2-expressing cells by increasing the amount of spike proteins in the organism [147,148]. Mechanistic trial results suggested a possible approach to infection prevention through the binding of Lf to HSPG on host cell surfaces, thereby preventing the binding of viral particles to this receptor [149]. Lf has also shown a capacity to adhere to anionic particles (e.g., viral particles) due to the cationic nature of its surface, inhibiting the entry of the virus into host target cells [150]. This involvement of Lf in the immune system suggests that it might be useful as an active agent in the prevention and treatment of COVID-19 [144,151]. 

In a preliminary clinical trial [67], patients with COVID-19 who were administered liposome-encapsulated bLf orally (1 g/day) and intranasally (16 mg/day) had significantly lower levels of IL-6 and D-dimers, considered therapeutic targets in COVID-19; they also exhibited significantly faster SARS-CoV-2 RNA negative conversion in comparison to controls receiving standard treatment with hydroxychloroquine and lopinavir/darunavir alone [152,153]. These results may indicate a possible gradual elimination of the virus, consistent with observations of a tendency towards remission of many symptoms (e.g., myalgia, arthralgia, ageusia, and anosmia) after 30 days of bLf treatment. The sole adverse effect was gastrointestinal discomfort in some participants, but it did not require interruption of the treatment [67]. In regard to safety, the tolerable daily dose of bLf established by the U.S. Food and Drug Administration ranges between 100 mg and 3 g [154].

Serrano et al. administered 32 mg/10 mL bLf plus 12 mg vitamin C to patients isolated and treated at home in four or six daily doses for 10 days and observed a reduction in myalgia, headaches, and fatigue after 5 days of treatment, with no noteworthy adverse effects [68]. Similar results were obtained in a randomized clinical trial that explored the effects of adding Lf at 200 mg/day or 400 mg/day to standard treatment alone. Higher hemoglobin levels and leukocyte (lymphocyte and neutrophil) and platelet counts were recorded in those receiving 400 mg/day in comparison to those receiving 200 mg/day and controls. Clinically relevant improvements were also observed in fever, coughing, headache, loss of the sense of taste, fatigue, and gastrointestinal discomfort, but differences did not reach statistical significance [69]. In other randomized clinical trial, no differences were observed between lactoferrin (800 mg) and placebo in the primary outcomes, the proportion of deaths, ICU admissions, and discharges, or the National Early Warning Score 2 [72].

The effects of Lf on COVID-19 disease have also been explored in an exclusively outpatient setting, comparing outcomes of standard treatment (ibuprofen, paracetamol, cortisone, or azithromycin) plus bLf with those of standard treatment alone. The bLf dose was 400 mg/day in asymptomatic patients, 600 mg/day in those with mild symptoms, and 1 g/day in those with moderate symptoms. Results obtained showed that the time to SARS-CoV-2 RNA negative conversion was 37.5% lower in patients treated with bLf. It was also observed that the reduction in symptoms was more marked with older age, possibly attributable to the hormonal influence on Lf production, which may increase the protective effect in this population [70,155]. In contrast, a randomized clinical trial in healthcare professionals found no significant difference in the frequency, duration, or symptoms of the disease between those receiving oral supplementation with 600 mg/day of bLf and those receiving placebo [71]. However, it should be noted that studies in the outpatient setting are limited by their reliance on the self-administration of treatments and the self-reporting of symptoms. 

There has been little research on the therapeutic potential of Lf in patients with COVID-19, but greater evidence is available on its effect in patients with other respiratory diseases [156]. Clinical trials have described a lower likelihood of respiratory system infection with LF supplementation and have supported its preventive potential, even in infant populations, and its therapeutic usefulness to reduce the duration of disease and mitigate symptoms [157,158,159]. These promising outcomes suggest the need for further in-depth investigations into the efficacy of this biomolecule to prevent and/or treat COVID-19.

### 2.5. Natural Products Found in Food as Supplements in SARS-CoV-2 Infection

#### 2.5.1. Curcumin

Curcumin, the active compound in turmeric, has attracted attention for its potential therapeutic properties in various diseases. Curcumin is well-known for its strong anti-inflammatory properties. COVID-19 can trigger a hyperinflammatory response, often referred to as a “cytokine storm”, which is responsible for severe complications like acute respiratory distress syndrome (ARDS). Curcumin might help modulate the immune response and reduce inflammation. In this sense, curcumin, which is used as a health food, exhibits anti-inflammatory effects by suppressing the activation of the transcription factor nuclear factor-kappa B (NF-κB) [73,160].

Several clinical trials have been conducted to evaluate the effectiveness of curcumin (cR) in COVID-19 patients. Kishimoto et al. evaluated the effect of 7-day oral intake of cR (360 mg twice daily). Within 5 days of COVID-19 diagnosis patients were randomly assigned to a placebo or cR group in a double-blind manner. The results showed that the relative suppression of event rates and antipyretic medications taken, and significant decrease of subclinical body temperature support the anti-inflammatory effects of cR in asymptomatic or mildly symptomatic patients with COVID-19 [73]. In this sense, Ahmadi et al. explored in a double-blind, randomized clinical trial the nanocurcumin’s effect on the clinical manifestations of patients hospitalized with mild-to-moderate COVID-19. The study suggest that 40 mg of nanocurcumin has a potentiating anti-inflammatory effect when combined with standard COVID-19 treatment, helping the recovery from the acute inflammatory phase of the disease in hospitalized patients with mild-to-moderate disease severity [77]. Similar studies by Asadirad et al. and Sadeghizadeh et al. also showed that C-reactive protein (CRP) and erythrocyte sedimentation rate (ESR) levels significantly decreased from baseline in COVID-19 patients treated with the nano-curcumin. Furthermore, blood levels of D-dimer, CRP, serum ferritin, ESR, and inflammatory cytokines including IL-6, IL-8, and IL-10 decreased more significantly in the nano-curcumin-treated group after 14 days. Additionally, the nano-curcumin group showed significant improvements in chest CT scores, oxygen saturation levels, and hospitalization duration [74,76]. In addition, Fessler et al. conducted a RCT in adults who had previously been diagnosed with COVID-19 and subsequently received a primary series of monovalent vaccine doses. A total of 500 mg of curcumin capsules twice daily for four weeks were administrated. The intake of curcumin confers anti-inflammatory activity and may be a promising prophylactic nutraceutical strategy for COVID-19. These results suggest that 4 weeks of curcumin supplementation resulted in significantly lower concentrations of proinflammatory cytokines in adults who recovered from COVID-19 infection and were subsequently vaccinated [75].

#### 2.5.2. Luteolin

Luteolin, a flavonoid found in various fruits, vegetables, and herbs, has been investigated for its potential role in combating COVID-19 due to its diverse biological activities. Transient anosmia is often present in the acute phase of Severe Acute Respiratory Syndrome Coronavirus 2 (SARS-CoV-2) infection, and it occurs most commonly in patients with mild or moderate COVID-19 [161,162,163]. Although the pathogenesis of COVID-19 olfactory dysfunction has not been fully elucidated, neuroinflammation is thought to play a critical role [164]. Few evidence-based therapies are available for chronic olfactory dysfunction after COVID-19. In this sense, Di Stadio et al. investigated the relative efficacy of olfactory training alone, co-ultramicronized palmitoylethanolamide with luteolin (um-PEA-LUT, an anti-neuroinflammatory supplement) alone, or combined therapy for treating chronic olfactory dysfunction from COVID-19. Their results showed that olfactory training plus a once-daily um-PEA-LUT resulted in greater olfactory recovery than either therapy alone in patients with long-term olfactory function due to COVID-19 [78]. In this sense, De Luca et al. investigated whether treatment with palmitoylethanolamide and luteolin (PEA-LUT) leads to improvement in the quantitative or qualitative measures of olfactory dysfunction or relief from mental clouding in patients affected by long COVID-19. They concluded that in patients with long COVID-19 and chronic olfactory loss, a regimen including oral PEA-LUT and olfactory training ameliorated olfactory dysfunction and memory [80]. Similar results were obtained by Di Stadio et al. when investigating the recovery of olfactory function in patients treated with PEA-LUT oral supplements plus olfactory training versus olfactory training plus placebo. Their results showed among individuals with olfactory dysfunction post-COVID-19, combining PEA-LUT with olfactory training resulted in greater recovery of smell than olfactory training alone [79].

#### 2.5.3. Ginger

Ginger (zingiber officinale) is a key spice and an herbal medicine that is widely used. Suppressing synthesis of some of Proinflammatory cytokines, such as interleukin-1, interleukin-8, and tumor necrosis factor alpha (TNF-alpha), are some of the effects of ginger. It also suppresses responses derived from T-helper1 activity [165,166], for this reason it has been explored for its potential role in managing COVID-19 due to its anti-inflammatory, antiviral, and immune-boosting effects. In this sense, Mesri et al. conducted a clinical trial with 100 suspected COVID-19 outpatients. The participants were allocated randomly to two groups of 50 members. The intervention group received concurrent *Zingiber officinale* (Tablet Vomigone 500 mg II tds) and Echinacea (Tablet Rucoldup I tds) for seven days in addition to the standard treatment. The control group only received the standard treatment (Hydroxychloroquine). The results showed that the improvement level in coughing, dyspnea, and muscle pain was higher in the intervention group (*p* value <0.05) [81]. A similar study was conducted by Singh et al. to assess the efficacy and safety of Ashwagandha [*Withania somnifera* (L.) Dunal] tablet and Shunthi (Zingiber officinale Roscoe) capsule in mild and moderate COVID-19 compared to conventional standard care. The results showed that these compounds can effectively reduce the duration of clinical recovery and improve time for viral clearance in mild and moderate COVID-19. These interventions were observed to be safe and well-tolerated during the duration of the trial [82]. Ginger shows promise as a complementary therapy for COVID-19 due to its antiviral, anti-inflammatory, antioxidant, and immune-modulating properties. While the current evidence is mostly based on in vitro studies, animal models, and traditional uses, ginger could be beneficial in managing COVID-19 symptoms or supporting recovery.

#### 2.5.4. Allicin

Allicin is one of the most common sulfenic acids found in garlic and has powerful bioactive properties. It is the compound that gives garlic its typical smell and flavor. The properties of this compound include cardioprotective, antimicrobial, antitumor, anti-inflammatory, and cholesterol-lowering properties [167,168]. Among the antimicrobial properties of this compound are those with antiviral potential against viral agents such as influenza A and B, cytomegalovirus, and HIV, among others [169,170].

Several studies have shown the immunomodulatory potential of this compound in COVID-19 disease. An in vitro study carried out with SARS-CoV-2-infected Vero E6 and Calu-3 cells showed that treatment of these cell lines with biocompatible doses of allicin led to a 60–70% decrease in viral RNA and infectious viral particles [171]. A randomized, placebo-controlled clinical trial involving 66 patients (33 experimental and 33 placebo) showed how allicin supplementation (L-cysteine/90 mg/kg), administered three times daily for two weeks, could significantly influence the improvement of signs and symptoms of SARS-CoV-2 infection. In this study, patients in the experimental group showed improvement in the presentation of cough, dyspnea and myalgias, as well as progress in lung involvement as measured by imaging tests (computed tomography) [83].

#### 2.5.5. Magnesium

Among the essential minerals at a physiological level, magnesium is a fundamental nutrient with a well-recognized role. In recent times, it has become clear that in many populations there is a nutritional deficiency of this mineral, which increases the risk of developing certain physical and mental illnesses [172,173]. Several studies have highlighted the importance of this mineral in pathologies such as obesity, type 2 diabetes mellitus and Duchenne muscular dystrophy [174]. Furthermore, magnesium plays an important role in the regulation of various immune functions of the immune system, both innate and adaptive, as well as in the regulation of acute and chronic inflammatory processes [175,176].

Thus, several studies have highlighted the importance of this mineral in the context of SARS-CoV-2 infection. Thus, in a clinical trial in hospitalized COVID-19 patients, vitamin D and magnesium supplementation therapy was used to manage the disease. After treatment of 17 patients with the combined therapy (17 experimental and 26 control patients), it was found that those who had received the supplementation therapy had an 87% lower risk of requiring oxygen therapy and an 87% lower risk of requiring intensive care [84]. Rostami et al. conducted a randomized clinical trial with 60 patients (30 experimental and 30 placebo) with moderate or severe COVID-19 disease. Patients in the experimental group were supplemented with 300 mg magnesium daily. The results showed that patients with additional magnesium supplementation therapy had lower oxygen therapy requirements, with improvements in oxygen saturation [85]. 

#### 2.5.6. Zinc

Zinc is the second most abundant trace element in the body after iron. Its functions include contributing to protein structure, catalyzing enzyme activities, and regulating gene expression. Moreover, zinc is vital to the body’s antioxidant defense system, mainly due to its role in the function of superoxide dismutase, a key enzyme that safeguards cells against oxidative damage. It also has important immunological functions, such as the differentiation, maturation and action of NK cells, as well as the production of IFN-γ and IL-12. It is present in a wide variety of foods, such as beef, poultry, seafood and cereals. This supplement is often used in various pathologies and deficiency states, such as zinc deficiency, diarrhea, age-related macular degeneration, upper respiratory infections, wound healing, and HIV infection [177,178]. 

Even taking into account the effect of this trace element at the immunological level, the use of this trace element in COVID-19 in various clinical trials has yielded inconclusive results. For example, a non-randomized clinical trial in healthy patients (n = 113) used zinc supplementation (20 mg/day) for 20 weeks. The results of this study showed a lower rate of infection in the experimental arm, with 15% SARS-CoV-2 infection in the control group and 0% in the experimental group. In this study, it is important to note that serum zinc levels were not measured and it was not specified whether the control group used a placebo [86]. On the other hand, Patel et al., conducted a randomized double-blind clinical trial with 33 hospitalized patients with a diagnosis of COVID-19 and an oxygen saturation of 94% or lower. Patients were treated for 7 days with 0.24 mg/kg/day of elemental zinc or placebo in the control group. Although the study had to close the patient recruitment process due to public health concerns, the results highlighted worse outcomes in terms of patient outcome in the experimental group compared to the control [87].

## 3. Conclusions

Supplementation with vitamin D, antioxidant vitamins (A, E, or C), melatonin, lactoferrin, and natural products found in food (curcumin, luteolin, ginger, allicin, magnesium, and zinc) may play a beneficial role in COVID-19 treatment, largely based on their immunomodulatory properties. The addition of these biomolecules to standard COVID-19 treatments has been associated with clinical improvements; however, only a limited number of studies have been published to date, and further research is needed to fully elucidate their usefulness. 

## Figures and Tables

**Table 1:** Different properties of biomolecules towards COVID-19 disease.

Biomolecule	Methodology	Main Findings	Reference
Vitamin D	Consensus document between professionals	Consistence evidences that stablish and association between vitamin D levels and poor COVID-19 outcomes. On the other side, the low vitamin D status in COVID-19 patients might also reflect reverse causality. Vitamin D supplementation might have a positive role in COVID-19 prevention.	Bilezikian et al., 2022 [21]
	Review	Most of the known conditions (aging and sex) and endocrine comorbidities (diabetes mellitus, obesity and body composition…), which associate with severe outcomes of COVID-19 that are characterized or caused by low VD levels.	Giustina, 2021 [22]
	Meta-analysis	Low vitamin D levels are related to an increased risk of severe COVID-19 infection, requiring admission to intensive care units or mortality, as well as a higher susceptibility to SARS-CoV-2 infection and related hospitalization.	Chiodini et al., 2021 [23]
	Retrospective cohort study	Very high incidence of hypocalcemia in COVID-19 patients and it predicts the need for hospitalization.	Di Filippo et al., 2020 [24]
	Retrospective cohort study	High prevalence of hypocalcemia in COVID-19 patients. It occurred in the context of marked hypovitaminosis D not adequately compensated by secondary hyperparathyroidism.	Di Filippo et al., 2021 [25]
	Systematic review and meta-analysis	Various studies with around 2 million adults suggest that low levels of vitamin D increases susceptibility to COVID-19 and severe COVID-19.	Dissanayake et al., 2022 [26]
	Retrospective, observational analysis	SARS-CoV-2 positivity is strongly and inversely associated with vitamin D levels.	Kaufman et al., 2020 [27]
	Analytic study	No direct relationship between vitamin D status, sun exposure, and SARS-CoV-2.	Ferrari et al., 2021 [28]
	Phase 3 open label randomized controlled trial.	Implementation of a population level test and intervention approach to vitamin D supplementation was not associated with a reduction in risk of all cause acute respiratory tract infection or COVID-19.	Jolliffe et al., 2022 [29]
	Systematic review and meta-analysis	An insufficient level of vitamin D increased hospitalization and mortality from COVID-19. There was a positive association between vitamin D deficiency and the severity of the disease.	Pereira et al., 2022 [30]
	Review of observational studies	Vitamin D deficiency is associated with greater severity of COVID-19 infection.	Wang et al., 2022 [31]
	Retrospective observational clinical trial	The study showed that 59% of sample patients that required hospitalization for severe COVID-19 pneumonia were vitamin D deficient. Presence of vitamin D deficiency on hospital admission is associated with COVID-19 mortality.	De Smet et al., 2021 [32]
	Analytical study	COVID-19 infection severity is associated with a significant decrease in vitamin D and its metabolites, with a significant increase in ACE2, Il-6, and NLR. Higher levels of vitamin D and its metabolites are protective against severe infection.	Khojah et al., 2022 [33]
	Longitudinal descriptive study	People with chronic conditions (obesity, hypertension, chronic obstructive pulmonary disease, and diabetes) combined with vitamin D deficiencies could be patients in high risk for infection and poorer outcomes. Vitamin D supplementation may assist in reducing risk of severe disease from COVID-19 particularly for individuals with pre-existing conditions.	Baxter et al., 2022 [34]
	Retrospective cohort study	Low vitamin D concentrations have a relationship with adverse clinical outcomes of COVID-19 infection, namely hospitalization and mortality.	Seal et al., 2022 [35]
	Analytical study	Vitamin D deficiency is associated with COVID-19 mortality. Low vitamin D may contribute to a pro-inflammatory and pro-thrombotic state, increasing the risk for adverse COVID-19 outcomes.	Vanegas-Cedillo et al., 2022 [36]
	Retrospective cohort study	Patients with vitamin D deficiency had a significantly higher mortality risk than those without vitamin D deficiency.	Neves et al., 2022 [37]
	Analytical study	Vitamin D deficiency was more frequently in male patients and in those affected by severe COVID-19. Patients with low levels of vitamin D and diabetes mellitus, as well those with low levels of vitamin D and overweight, were more frequently affected by a severe disease with worse inflammatory response and respiratory parameters.	di Filippo et al., 2022 [38]
	Mendelian randomization study	The authors did not observe an association between vitamin D levels and COVID-19 susceptibility, severity, or hospitalization.	Butler-Laporte et al., 2021 [39]
	Randomized controlled trial	The number of days on respiratory support did not show a statistically significant difference, even though the trial lacked sufficient power for the main outcome.	Domazet Bugarin et al., 2023 [40]
	Randomized controlled trial	Vitamin D did not contribute to a shorter recovery time in patients who still tested positive by RT-PCR on the 14th day.	Abroug et al., 2023 [41]
	Randomized controlled trial	The intervention group showed a more substantial reduction in the pneumonia severity index from enrollment to discharge. Additionally, patients with C-reactive protein levels greater than 30 mg/L also experienced a significant decrease.	Dilokpattanamongkol et al., 2024 [42]
Vitamin A	Triple-blind controlled clinical trial	After 10 days, post-intervention in the experimental group, vitamin A supplementation demonstrated showed significantly greater decreases such as fever, body ache, weakness and fatigue, paraclinical symptoms, white blood cell count, and C-reactive protein showed.	Rohani et al., 2022 [43]
	Pilot randomized controlled clinical trial	Intramuscular vitamin A administration for two weeks was not significantly different between the two groups for either clinical response or time to clinical response. There were also no significant differences in terms of need for mechanical ventilation, time to hospital admission, or hospital death.	Somi et al., 2022 [44]
	Analytical study	Results show a correlation between serum retinol level and severe COVID-19 infection. Despite the continued use of Favipiravir and hydroxychloroquine, which inhibit retinol metabolism, and the presence of vitamins, including vitamin A, in the nutritional formulas administered, the serum retinol level was significantly lower in severe COVID-19 cases.	Sarohan et al., 2022 [45]
Vitamin E	Open, quasi-experimental, analytical, prospective, and longitudinal (before–after) study	Treatment with antioxidant supplements, such as vitamins C and E, among others, plus pentoxifylline (Px) reversed low levels of LPO and inflammatory mediators (IL-6, CRP, and PCT). Improving survival scores including SOFA, Apache II, SAPS II, COVIDGRAM, and GCS.	Chavarría et al., 2021 [46]
	Randomized controlled clinical trial	Significant changes were detected in serum levels of vitamins, ESR, CRP, IL6, TNF-a, and SOFA score after intervention compared with the control group. The prolonged hospitalization rate to more than 7 days was significantly lower in the intervention group. However, the effect on mortality showed no significant difference.	Beigmohammadi et al., 2021 [47]
Vitamin C	Systematic review and meta-analysis of Randomized controlled clinical trials	VC treatment didn’t reduce mortality, ICU length of stay, hospital length of stay or need for invasive mechanical ventilation.	Rawat et al., 2021 [48]
	Observational study	VC plasma concentration in patients with COVID-19 was almost 5-fold lower than that in healthy volunteers. Thus, supplementation is considered highly essential.	Xing et al., 2021 [49]
	Randomized controlled clinical trial	VC supplementation resulted in a higher survival duration. There was a linear association between the number of days of vitamin C intake and survival duration. Level of serum K^+^ was lower in the patients compared with the control group, which also seems to be related to longer survival.	Majidi et al., 2021 [50]
	Open-label, randomized controlled clinical trial	After 3 days of hospitalization, patients receiving high doses of VC in addition to conventional treatment had lower temperature and higher peripheral capillary oxygen saturations, but the length of hospitalization was also higher.	JamaliMoghadamSiahkali et al., 2021 [51]
	Retrospective study	VC supplementation in addition to conventional treatment in ICU patients was associated with a lower incidence of thrombosis.	Al Sulaiman et al., 2021 [52]
	Randomized controlled clinical trial	Patients treated with VC plus standard treatment had higher oxygen saturation and lower respiratory rate. They also showed less lung involvement measured on chest CT.	Tehrani et al., 2022 [53]
	Randomized controlled clinical trial	Patients taking VC showed no better results than those taking melatonin or pacebo.	Fogleman et al., 2022 [54]
	Randomized controlled clinical trial	Patients treated with VC achieved a 50% reduction in a shorter time than patients with standard treatment.	Thomas et al., 2021 [55]
	Open label, randomized clinical trial	Patients treated with a combination of Zn+VC had higher levels of IgG anti SARS-CoV-2 and higher levels of transitional B cells	Quek et al., 2022 [56]
	Single-blind randomized controlled trial	Patients with long COVID treated with VC+L-arginine for 28 days improved walking performance, muscle hand strength, endothelial-vascular function and fatigue.	Tosato et al., 2022 [57]
	Randomized controlled trial	After 28 days of supplementation with l-arginine plus vitamin C, there was a significant increase in serum l-arginine concentrations and the l-arginine/ADMA ratio compared to the placebo group.	Calvani et al., 2023 [58]
Melatonin	Randomized controlled clinical trial	RCT in healthcare workers with high exposure to SARS-CoV-2 using oral melatonin (2 mg/day for 12 weeks) prophylactically to prevent SARS-CoV-2 infection. The results showed that prophylactic oral melatonin administration was not effective in preventing SARS-CoV-2 infection in healthcare workers.	García-García et al., 2022 [59]
	Randomized controlled clinical trial	The mortality rate observed in patients treated with melatonin (test group) was significantly lower (67%) than in the control group (94%). Likewise, the need for invasive mechanical ventilation (IMV) was significantly lower in the test group (51.4%) than in the control group (70.9%), like the mean number of days of hospitalization (15 days, test group; 21 days, control group).	Ameri et al., 2022 [60]
	Multicenter retrospective observational study	This observational restrospective study failed to observe a reduction in mortality in adult patients hospitalized for COVID-19 who were treated with a mean melatonin dose of 2.1 mg for a mean of 15 days.	Sánchez-Rico et al., 2022 [61]
	Randomized controlled clinical trial	RCT using high doses of melatonin (21 mg/day) as adjuvant therapy in patients intubated for COVID-19. Although C-reactive protein (CRP) levels decreased significantly in the melatonin-treated group, indicating a decrease in the inflammatory response, they did not observe significant differences in the mortality rate, nor in the duration of mechanical ventilation in these patients with respect to the control group.	Alizadeh et al., 2022 [62]
	Randomized controlled clinical trial	The use of oral melatonin at low doses (3 mg, 3 times/day) in patients hospitalized for COVID-19 (test group) had a significant effect (*p* < 0.05) on the decrease of cough, dyspnea, fatigue, and CRP levels compared to the control group (conventional treatment).	Farnoosh et al., 2022 [63]
	Randomized controlled clinical trial (RCT)	The administration of low doses of oral melatonin (3 mg before bedtime) in patients hospitalized for COVID-19 showed a significant improvement in the quality of sleep (*p* < 0.001), as well as in the oxygen saturation levels (*p* = 0.003) of these patients.	Mousavi et al., 2021 [64]
	Randomized controlled clinical trial	The results showed a significant decrease (*p* < 0.05) in the occurrence of thrombosis and sepsis in patients in the test group (melatonin), as well as a significantly lower mortality rate (*p* < 0.05).	Hasan et al., 2022 [65]
	Randomized controlled clinical trial	They observed that patients treated with oral vitamin C (1000 mg/day) did not obtain a significant improvement in symptomatology with respect to the placebo group. However, the group treated with oral melatonin (10 mg/day) obtained better results with respect to symptomatology improvement (*p* < 0.003) and quality of life improvement with respect to the vitamin C group and placebo group (*p* < 0.005).	Fogleman et al., 2022 [54]
	Randomized clinical trial	Melatonin significantly improved clinical status (*p* < 0.05) (mortality rate, number of days to hospital discharge and time to clinical status improvement) with a safe profile in patients with severe COVID-19 pneumonia.	Ameri et al., 2023 [66]
Lactoferrin	Randomized controlled clinical trial	The results showed that patients with COVID-19 treated with oral liposomal bovine lactoferrin (bLf) (1000 mg/day) and intranasal bLf (16 mg/day) have an earlier SARS-CoV-2 RNA negative conversion. Moreover, this treatment achieved a decrease in IL-6 and D dimers and improved the symptoms.	Campione et al., 2021 [67]
	Prospective observational study	Patients with COVID-19 were treated at home with 32 mg of Lf and 12 mg of vitamin C (four or six doses per day for 10 days). The authors observed a complete recession of symptoms after 5 days of treatment.	Serrano et al., 2020 [68]
	Randomized controlled clinical trial	After administration of Lf at a dose of 400 mg/day, the authors observed an increase in haemoglobin, white blood cell and platelet counts. In addition, improvements in symptoms such as cough, headache, fever and loss of taste were observed in patients with COVID-19.	Algahtani et al., 2021 [69]
	Retrospective observational study	The addition of bLf at doses of 400 mg/day in asymptomatic, 600 mg/day in mild symptomatic and 1000 mg in moderate symptomatic patients accelerated SARS-CoV-2 RNA negativation. In addition, symptom attenuation was observed in elderly patients.	Rosa et al., 2021 [70]
	Randomized controlled clinical trial	A comparison was made between daily supplementation with 600 mg of enteral bLF and a placebo over 90 days. bLF had no significant impact on the time to symptomatic infection. Additionally, there were no notable differences in secondary outcomes such as severity, frequency, and duration of symptomatic infection.	Navarro et al., 2023 [71]
	Randomized controlled clinical trial	No differences were observed between lactoferrin (800 mg) and placebo in the primary outcomes: the proportion of deaths or ICU admissions, the proportion of discharges, or the National Early Warning Score 2.	Matino et al., 2023 [72]
Curcumin	Randomized controlled clinical trial	Curcumin had anti-inflammatory effects in asymptomatic or mildly symptomatic COVID-19 patients, as evidenced by a relative reduction in event rates, fewer instances of antipyretic medication use, and a significant decrease in subclinical body temperature.	Kishimoto et al., 2024 [73]
	Randomized controlled clinical trial	A significant reduction in C-reactive protein (CRP) and erythrocyte sedimentation rate (ESR) levels from baseline in the nano-curcumin group were observed by day 7. After 14 days, levels of D-dimer, CRP, serum ferritin, ESR, and inflammatory cytokines decreased more markedly in the nano-curcumin-treated group.	Sadeghizadeh et al., 2023 [74]
	Controlled clinical trial	These results suggest that 4 weeks of curcumin supplementation resulted in significantly lower concentrations of proinflammatory cytokines in adults who recovered from COVID-19 infection and were subsequently vaccinated.	Fessler et al., 2023 [75]
	Controlled clinical trial	A significant difference in the expression of IFN-γ, IL-1β, and IL-6 was reported between the nano-curcumin and control groups.	Asadirad et al., 2022 [76]
	Randomized controlled clinical trial	The findings indicate that nanocurcumin amplifies the anti-inflammatory effects when used alongside standard COVID-19 treatment, supporting recovery from the acute inflammatory phase in hospitalized patients with mild-to-moderate disease severity.	Ahmadi et al., 2023 [77]
Luteolin	Randomized controlled clinical trial	Their results showed that olfactory training plus once daily with palmitoylethanolamide and luteolin resulted in greater olfactory recovery than either therapy alone in patients with long-term olfactory function due to COVID-19	Di Stadio et al., 2023 [78]
	Randomized controlled clinical trial	The intervention group demonstrated significantly greater improvements in olfactory threshold, discrimination, and identification scores compared to the control group.	Di Stadio et al., 2022 [79]
	Longitudinal study	Treatment with palmitoylethanolamide and luteolin leads to improvement in the quantitative or qualitative measures of olfactory dysfunction or relief from mental clouding in patients affected by long COVID-19.	De Luca et al., 2022 [80]
Ginger	Randomized controlled clinical trial	The intervention group experienced greater improvement in coughing, dyspnea, and muscle pain. However, there was no significant difference between the two groups regarding other symptoms.	Mesri et al., 2021 [81]
	Randomized controlled clinical trial	The results showed that theses compunds, *Withania somnifera* (L.) and Zingiber officinale Roscoe, could effectively reduce the duration of clinical recovery and improve time for viral clearance in mild and moderate COVID-19.	Singh et al., 2023 [82]
Allicin	Randomized controlled clinical trial	Allicin supplementation (L-cysteine/90 mg/kg), administered three times daily for two weeks, could significantly influence the improvement of signs and symptoms of SARS-CoV-2 infection (cough, dyspnea and myalgias).	Yaghoubian et al., 2021 [83]
Magnesium	Clinical trial	After the treatment of 17 patients with the combined therapy, it was found that those who had received the supplementation therapy had an 87% lower risk of requiring oxygen therapy, and an 87% lower risk of requiring intensive care.	Tan et al., 2020 [84]
	Clinical trial	The results showed that patients with additional magnesium supplementation therapy had lower oxygen therapy requirements, with improvements in oxygen saturation.	Rostami et al., 2024 [85]
Zinc	Clinical trial	The results of this study showed a lower rate of infection in the experimental arm, with 15% SARS-CoV-2 infection in the control group and 0% in the experimental group.	Margolin et al., 2021 [86]
	Randomized controlled clinical trial	The results highlighted worse outcomes in terms of patient outcome in the experimental group compared to the control.	Patel et al., 2021 [87]
